# Random PCR-based nanopore whole-genome sequencing enables pre-positivity detection of fungal bloodstream infections

**DOI:** 10.1128/spectrum.00478-26

**Published:** 2026-08-21

**Authors:** Isato Yoshioka, Momotaka Uchida, Yoko Kusuya, Saho Shibata, Teppei Arai, Takashi Yaguchi, Akira Watanabe, Hiroki Takahashi

**Affiliations:** 1 Medical Mycology Research Center, Chiba University 12737, Chiba, Japan; 2 Biological Resource Center, National Institute of Technology and Evaluation 13594, Kazusakamatari, Kisarazu, Japan; 3 Faculty of Science, Chiba University 12737, Chiba, Japan; 4 Molecular Chirality Research Center, Chiba University 12737, Chiba, Japan; 5 Plant Molecular Science Center, Chiba University 12737, Chiba, Japan; 6 Synergy Institute for Futuristic Mucosal Vaccine Research and Development, Chiba University 12737, Chiba, Japan; National Institute for Communicable Diseases, Johannesburg, Gauteng, South Africa

**Keywords:** real-time diagnosis, *Candida* species, fungal bloodstream infections, whole-genome sequencing

## Abstract

Fungal bloodstream infections (BSIs), particularly those caused by *Candida* species, represent a major clinical challenge associated with substantial morbidity and mortality. Timely initiation of appropriate antifungal therapy is critical for patient survival; however, conventional diagnostic methods rely on blood culture positivity and downstream identification procedures, resulting in delays that limit early, targeted treatment. In this study, we developed and validated an optimized workflow for rapid identification of fungal pathogens directly from blood culture samples collected prior to positivity in automated blood culture systems using real-time nanopore sequencing. The workflow consists of selective depletion of host genomic DNA, microbial DNA extraction, and species identification via random PCR-based whole-genome amplification, followed by sequencing on the MinION platform and real-time taxonomic classification using a custom database. Host DNA was effectively removed using saponin-mediated cell lysis and benzonase treatment, enabling enrichment of microbial DNA. Using this approach, species-level identification was achieved within 1.5 h from sequencing initiation, and accurate identification was possible with as few as 4,000 sequencing reads. The total turnaround time of the workflow was approximately 7 h. Validation using 48 clinical blood culture samples demonstrated high concordance with routine clinical diagnoses, and the method reliably detected both monomicrobial and polymicrobial infections, including fungal–fungal and fungal–bacterial co-infections. Genome-wide sequencing data enabled detection of candidate resistance-associated variants in clinically relevant genes. Overall, this workflow provides a clinically actionable solution for early diagnosis of fungal BSIs, with the potential to reduce diagnostic delays, inform antifungal selection, and improve patient outcomes.

**IMPORTANCE:**

Fungal bloodstream infections (BSIs) are serious and often fatal conditions that require prompt antifungal treatment. However, conventional diagnostic approaches depend on blood culture positivity and subsequent identification steps, leading to delays that can compromise patient outcomes. Here, we present a rapid sequencing-based workflow that enables direct identification of fungal pathogens from blood culture samples before they become positive. By combining host DNA depletion with real-time nanopore sequencing, our method delivers species-level results within hours and accurately detects both single-species and mixed infections. This capability is particularly important in clinical settings where early identification of fungal species can inform antifungal choice. Importantly, the workflow is compatible with existing laboratory infrastructure and requires only a limited number of sequencing reads, supporting routine use. By substantially shortening the time to actionable results, this approach has the potential to improve clinical decision-making, optimize antifungal therapy, and reduce morbidity and mortality from fungal BSIs.

## INTRODUCTION

Bloodstream infections (BSIs) caused by microorganisms, such as bacteria and fungi, are a major cause of morbidity and mortality in hospitalized patients (1). *Candida* species were reported as the fourth leading cause of nosocomial BSIs following coagulase-negative staphylococci, *Staphylococcus aureus*, and enterococci (2). Globally, *Candida* species continue to represent the most common cause of invasive fungal infections (3–6).

More than 17 *Candida* species, including *C. albicans* and various non-*albicans* species, have been identified as etiologic agents in humans (1, 7, 8). Over 90% of invasive *Candida* infections are attributed to five major species: *C. albicans*, *Nakaseomyces glabratus* (formerly *C. glabrata*), *C. parapsilosis*, *C. tropicalis*, and *Pichia kudriavzevii* (formerly *C. krusei*) (9–11). In addition, *Candidozyma* (formerly *Candida*) *auris* has emerged as a clinically significant pathogen (12) and is increasingly recognized as an important cause of nosocomial BSIs across Asia, Africa, the Americas, and Europe (13).

Treatment of *Candida* BSIs typically involves antifungal agents, including polyenes, echinocandins, azoles, and flucytosine (14). Echinocandins are currently recommended as the first-line treatment for moderately to severely ill patients with candidemia (14, 15), while fluconazole remains a viable option in certain clinical scenarios. However, antifungal resistance profiles vary among *Candida* species (16). Therefore, early and accurate species identification is critical for selecting appropriate antifungal therapy.

The gold standard for diagnosing BSIs is blood culture, followed by species identification and susceptibility testing (17). However, this process takes several days (18), delaying antifungal therapy and increasing mortality. Over half of patients receive antifungal treatment 24–48 h after the initial positive culture signal (19). Although rapid diagnostic platforms, such as T2Candida, FilmArray BCID, and PNA FISH (18, 20), exist, they are only applicable after blood culture positivity, limiting their use for early diagnosis.

Recent advances in nanopore sequencing technology have highlighted its potential for real-time microbial identification directly from clinical specimens (21). Building on this progress, Yonkus et al. (22) detected both fungal and bacterial pathogens, including *Candida* species, from bile duct cultures (22). Ohta et al. (23) developed an identification strategy targeting internal transcribed spacer (ITS) region sequences in sputum samples (23). However, no study has applied this approach to the early detection of fungal species in BSIs.

In this study, we developed a rapid and accurate diagnostic workflow for detecting fungal species in BSIs using 48 blood culture samples collected prior to positivity in automated culture systems. Our objective was to identify fungal pathogens at earlier time points to enable timely clinical intervention. To this end, we optimized a DNA extraction protocol that efficiently depletes human DNA in blood culture samples and generated a custom database for real-time species identification on the MinION platform. The protocol proved effective across a range of yeast pathogens, including but not limited to *Candida* species, underscoring its broader applicability. The total turnaround time of the entire workflow, including sample preparation, host DNA depletion, DNA extraction, library preparation, sequencing, and taxonomic analysis, was approximately 7 h. This approach substantially reduces turnaround time for species identification, facilitating earlier administration of targeted antifungal therapy. Collectively, our method holds promise for reducing diagnostic delays, improving clinical outcomes, and lowering mortality associated with fungal BSIs.

## MATERIALS AND METHODS

### Strains and culture conditions

Residual blood culture bottles (BACTEC; Becton, Dickinson and Company, Franklin Lakes, NJ, USA) were retrospectively collected from patients diagnosed with fungal BSIs. Each fungal strain isolated from blood culture was cultured on Sabouraud dextrose agar supplemented with chloramphenicol (BBL; Becton, Dickinson and Company) and identified based on ribosomal ITS sequences (24). *C. albicans* N508 (IFM 5740) and *C. neoformans* var. *grubii* CUH-1 (IFM 5807) were provided by the Medical Mycology Research Center, Chiba University with support in part by the National BioResource Project (NBRP), MEXT, Japan (https://nbrp.jp/). These fungal strains were used for the spike-in experiments. In addition, *Escherichia coli* CSH2 and *S. aureus* 2021-I1397 (25) were used as bacterial targets in the spike-in experiments. Fungal and bacterial strains were cultured on potato dextrose broth (PDB) and Luria–Bertani–Lennox (LBL) media at 37°C, respectively.

### Fungal DNA extraction from blood culture

Fungal DNA was extracted from 1 mL of blood culture following depletion of human DNA based on previously published methods (21, 26, 27) with modifications.

#### Red blood cell lysis

Three volumes of Red Blood Cell Lysis Solution (CLB; Promega, Madison, WI, USA) were added to the blood culture. The mixture was centrifuged at 3,000 × *g* for 10 min, and the supernatant was discarded. This step was repeated once.

#### Human cell lysis

The resulting pellet was resuspended in a volume of 20 mM Tris-HCl buffer (pH 8.0) containing 1 mM MgCl_2_ and 2.5% (wt/v) saponin equal to the initial blood culture volume. The suspension was centrifuged at 3,300 × *g* for 10 min, and the supernatant was discarded. This process was repeated once.

#### Depletion of human DNA

The pellet was resuspended in 1 mL of 20 mM Tris-HCl buffer (pH 8.0) with 1 mM MgCl_2_ and incubated at 37°C for 1 h with 1 μL of benzonase nuclease (Merck KGaA, Darmstadt, Germany). To terminate the reaction, 100 μL of 500 mM EDTA and 30 μL of 5 M NaCl were added. The mixture was centrifuged at 3,300 × *g* for 10 min, and 850 μL of supernatant was discarded.

#### Fungal cell lysis and DNA extraction

The pellet was resuspended in the remaining buffer, supplemented with 300 μL of lysis buffer and 30 μL of Proteinase K (Maxwell RSC Blood DNA Kit; Promega). The suspension was transferred to a Lysing Matrix D tube (MP Biomedicals, Irvine, CA, USA) and homogenized using a FastPrep-24 5G instrument (MP Biomedicals) for two 30-s cycles at 6.0 m/s. The samples were incubated at 70°C for 10 min. Fungal DNA was extracted using the Maxwell RSC Blood DNA Kit. Note that the initial blood culture volume used for sample S11 was 400 μL; all other samples used 1 mL.

### DNA purification and quantification

The extracted DNA was purified and concentrated by washing with a 1.2× AMPure XP bead (Beckman Coulter, Brea, CA, USA) and eluted with 15 μL of DNase-free water. An aliquot (~5 µL) of elution was used for DNA quantification by the Qubit dsDNA High-Sensitivity Assay Kit (Thermo Fisher Scientific, Waltham, MA, USA) on the Qubit 3.0 fluorometer (Thermo Fisher Scientific).

### Quantitative PCR (qPCR) assay to estimate the depletion of human genomic DNA

To confirm the depletion of human DNAs, quantitative PCR (qPCR) was performed on a LightCycler 96 Real-Time PCR System (Roche Diagnostics, Basel, Switzerland) with the KAPA SYBR Fast qPCR Kit (Universal qPCR) (KAPA Biosystems) under the following conditions: 95°C for 3 min (1 cycle); 95°C for 10 s; and 62°C for 30 s (40 cycles). A total of 20 ng of DNA extracted from sample S11 with or without benzonase treatment was used as template. The primers targeting the rDNA sequences of 18S (28) and 28S (29) were used for *Candida* species. The KAPA hgDNA Quantification 129 bp primer (KK4966; KAPA Biosystems, Wilmington, MA, USA) was used for human DNA.

### Spike-in analysis for DNA extraction efficiency

To validate the efficiency of the DNA extraction workflow, spike-in experiments were conducted using sample S11. Mock microbial communities consisting of *C. albicans*, *C. neoformans*, *E. coli*, and *S. aureus* were prepared and added to the sample prior to the DNA extraction. *C. albicans* and bacteria were cultured for approximately 12 h and *C. neoformans* for 3 days at 37°C with agitation at 200 rpm. Microbial growth was monitored by measuring the optical density at 600 nm (OD_600_) until it reached 5.0. Subsequently, each culture was combined and centrifuged to collect the cells. The resulting pellet was then mixed with 400 μL of blood culture sample S11 prior to DNA extraction. Three spike-in conditions with different microbial culture volumes were tested: Spike1 (1 mL for both fungi and bacteria), Spike2 (200 µL for both fungi and bacteria), and Spike3 (fungi: 1 mL; bacteria: 200 µL).

### MinION library preparation and sequencing

Nanopore sequencing was performed with rapid sequencing DNA–PCR Barcoding (SQK-RPB004; Oxford Nanopore Technologies, Oxford, UK) for samples S1–S12 and rapid sequencing DNA V14–barcoding (SQK-RBK114.24; Oxford Nanopore Technologies) for samples S13–S48.

#### Tagmentation and fragmentation

Fifteen nanograms of AMPure XP-purified DNA was tagmented with 2.5 μL of Fragmentation Mix (FRM; Rapid PCR Barcoding Kit, SQK-RPB004; Oxford Nanopore Technologies) in a final volume of 10 μL using nuclease-free water. The reaction was carried out at 30°C for 1 min with SQK-RPB004 or at 30°C for 1 min with SQK-RBK114.24, followed by 75°C for 1 min (SQK-RPB004) or 80°C for 2 min (SQK-RBK114.24). For samples with low DNA concentration (<2 ng/µL), 7.5 μL of the DNA solution was used directly as input for the tagmentation step.

#### PCR amplification

PCR was performed in a total reaction volume of 100 μL using the following thermal cycling program: 95°C for 3 min (initial denaturation); 25 cycles of 95°C for 15 s, 56°C for 15 s, and 65°C for 4 min; final extension at 65°C for 6 min.

#### Purification and quantification

PCR products were purified using 0.6 × AMPure XP beads and eluted in 14 μL of 10 mM Tris-HCl (pH 8.0) containing 50 mM NaCl (SQK-RPB004) or 15 μL of elution buffer supplied in the kit (SQK-RBK114.24). DNA concentrations were measured using the Qubit dsDNA Broad Range Assay Kit (Thermo Fisher Scientific).

### Construction of the custom database for the wf-metagenomics pipeline

The custom database was generated for use with the wf-metagenomics pipeline of EPI2ME v2.6.1 (https://github.com/epi2me-labs/wf-metagenomics) by using Kraken2 v2.1.2 (30) and Bracken v2.9 (31). In addition to 73,877 sequences included in PlusPFP-8, excluding Plasmid and UniVec sequences, the genomes of *C. parapsilosis* CDC317 (GCF_000182765.1), *C. tropicalis* MYA-3404 (GCF_000006335.3), *M. guilliermondii* ATCC 6260 (GCF_000149425.1), and *W. anomalus* NRRL Y-366-8 (GCF_001661255.1) were downloaded from National Center for Biotechnology Information (NCBI) and added to the database. TaxIDs were added to all FASTA file headers prior to database construction. In total, 73,881 sequences were incorporated into the custom database using kraken2-build with “--add-to-library.” The database was built by kraken2-build with “--max-db-size 16000000000” and subsequently bracken-build with “-l 1000.”

### MinION sequencing and real-time species identification

MinION sequencing was performed using either R9.4 or R10 flow cells for 12 (S1–S12) or for 36 samples (S13–S48), respectively. Approximately 100 fmol of prepared library was loaded onto the flow cell. For the single-sample run (sample S11), basecalling was conducted using Guppy v6.4.2 (https://nanoporetech.com/ja/software/other/guppy) for up to 3 h. In multiplexed sequencing runs, real-time basecalling and barcode demultiplexing were performed using Dorado v0.9.2+2634e9f (https://github.com/nanoporetech/dorado/). Concurrently, species identification was carried out using the wf-metagenomics pipeline with the custom database. Species identification from blood culture samples was based on results obtained during the first 1.5 h of sequencing. Sequencing was then continued for up to 17 h for 11 samples, 12 h for 24 samples, and 24 h for 12 samples. These extended data sets were used for downstream analyses. Sequencing quality and metrics were evaluated using NanoPlot v1.41.0 (32).

### Sequence analysis

Minimap2 v2.24-r1122 (33) with the option “secondary=no” was used to map the reads to the reference genomes of *N. glabratus* CBS 138 (GCA_000002545.2), *C. albicans* SC5314 (GCA_000182965.3), and *C. dubliniensis* CD36 (GCA_000026945.1). The depth and covered bases were calculated using samtools v1.17 (34) and CoverM v0.6.1 (35). The genome-wide coverage was visualized using pafr v0.0.2 (https://dwinter.github.io/pafr/articles/Introduction_to_pafr.html). The reads of sample S7 were mapped against the concatenated genome of *C. albicans* and *C. dubliniensis*. BLASTN v2.15.0 (36) with the nt database and taxonkit v0.15.1 (37) were performed for the species assignment.

Additional 18 *C. albicans* and 38 *N. glabratus* strains (Table S1) were obtained at short read archive under accession PRJNA432884, PRJNA361477, and PRJNA297263 (38–40). The reads were trimmed using fastp v0.20.1 (41). The filtered reads were mapped using BWA-MEM v0.7.17-r1188 (42). SNP analysis was performed by bcftools v1.21 (43). Briefly, the bam files were used for the mpileup command with “-B -Q5 --max-BQ 30 -I,” followed by the call command using “--ploidy 2” for *C. albican*s and “--ploidy 1” for *N. glabratus*. The vcf files were filtered by VCFtools v0.1.16 (44) with the options “--maf 0.05 --max-missing 1.” The fasta files were generated by vcf2phylip v2.9 (45). The SNP distance matrices were generated using snp-dists (https://github.com/tseemann/snp-dists). Phylogenetic trees were constructed using the multithreaded version of RAxML v8.2.1256, the GTRCAT model, and 1,000 bootstrap replicates (46). The phylogenetic trees were visualized using iTol v7.2.2 (47). The SNP annotation was performed by SNPEff v5.1d (48) with comparison to the reference sequences of *ERG11* (CAALFM_C500660CA) and *FKS1* (CAALFM_C102420CA) for *C. albicans* SC5314 and *ERG11* (GVI51_E04037) for *N. glabratus* CBS 138, respectively.

## RESULTS

### Optimized protocol for rapid diagnosis of *Candida* bloodstream infections

The infections of 48 patients involved eight fungal species spanning five genera and four families (Table 1); *Candida albicans* (*n* = 28), *C. parapsilosis* (*n* = 3), *C. dubliniensis* (*n* = 1), *C. tropicalis* (*n* = 1), *Meyerozyma guilliermondii* (formerly *C. guilliermondii*) (*n* = 1), *N. glabratus* (*C. glabrata*) (*n* = 10), *Cryptococcus neoformans* (*n* = 3), and *Wickerhamomyces anomalus* (*n* = 1).

**TABLE 1:** Samples of fungal bloodstream infections collected in this study

Family	Genus	Species	# of samples
*Debaryomycetaceae*	*Candida*	*albicans*	28
		*parapsilosis*	3
		*tropicalis*	1
		*dubliniensis*	1
	*Meyerozyma*	*guilliermondii*	1
*Saccharomycetaceae*	*Nakaseomyces*	*glabratus*	10
*Cryptococcaceae*	*Cryptococcus*	*neoformans*	3
*Wickerhamomycetaceae*	*Wickerhamomyces*	*anomalus*	1

**Fig 1:**
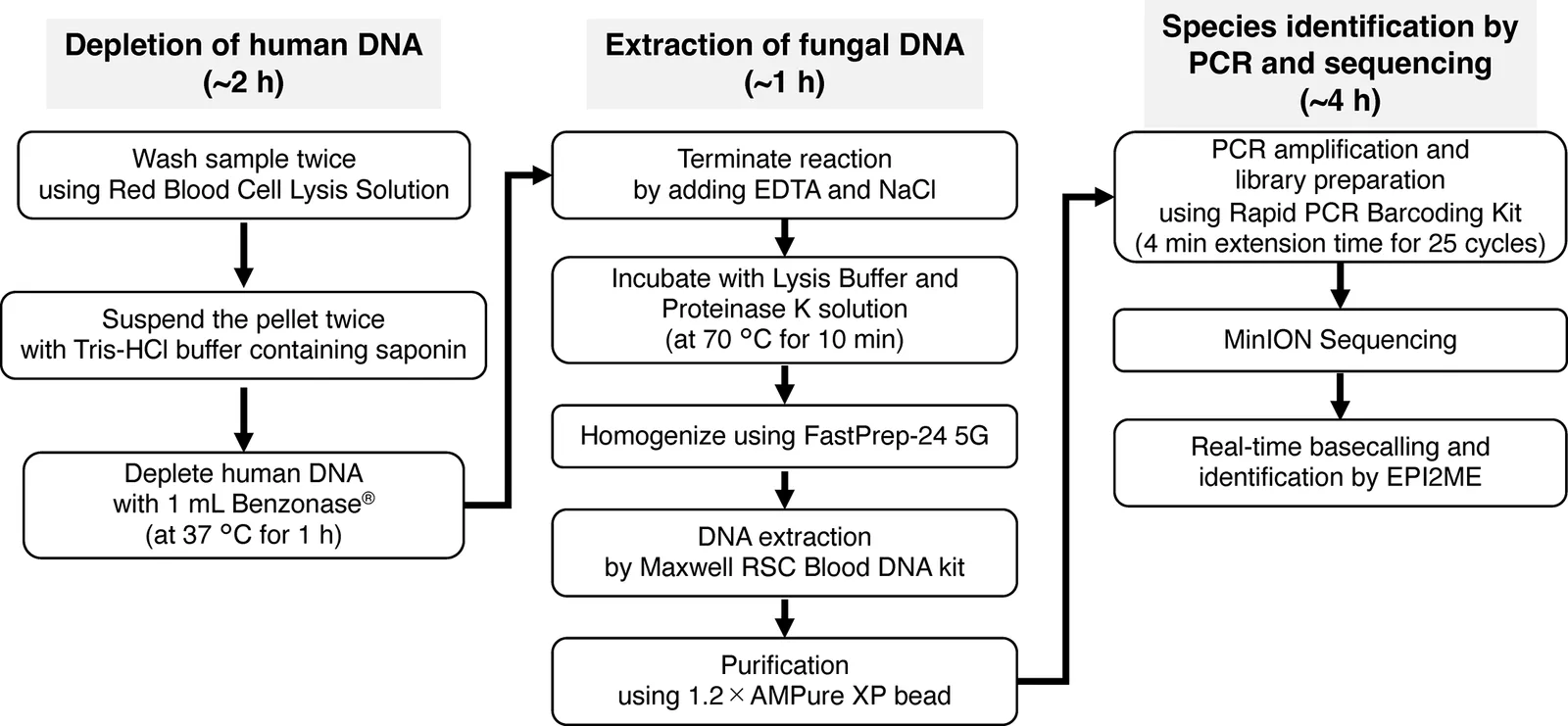
Overview of the optimized protocol for rapid diagnosis of fungal BSIs from blood culture. The workflow consists of three main steps: (i) depletion of human DNA (~2 h), (ii) extraction of fungal DNA (~1 h), and (iii) species identification via PCR and nanopore sequencing (~4 h).

To develop a rapid diagnostic method for fungal BSIs using MinION sequencing, we optimized the protocol (Fig. 1).

The wf-metagenomics pipeline enables real-time taxonomic identification. However, the prebuilt databases, such as PlusPFP-8 and EuPathDB46 (49), lack some species causing fungal BSIs in this study (Table S2). To enhance diagnostic accuracy, we constructed a custom database tailored for fungal BSI diagnosis.

Efficient removal of human DNA prior to fungal DNA extraction is critical for successful species-level identification. We evaluated the effectiveness of benzonase treatment using sample S11 clinically diagnosed with *N. glabratus* infection. The human DNA levels were markedly reduced to 0.21% of the original amount without compromising the yield of fungal DNA (Table 2). These results indicate that benzonase selectively degrades human DNA while preserving fungal DNA. Moreover, the combined use of red blood cell lysis and saponin treatment effectively lyses human cells without affecting fungal cells, enabling efficient and selective depletion of human DNA.

**TABLE 2:** Evaluation of the efficiency of human DNA extraction

Target	Gene	Ct value		Efficacy of depletion
		w/o benzonase	w benzonase	
Fungi	18S rRNA	10.83	10.71	1.1
	28S rRNA	9.56	9.32	1.2
Human	KAPA	23.61	32.51	2.1 × 10^−3^

To validate the efficiency of the DNA extraction workflow, spike-in experiments were conducted. Precultured fungi (*C. albicans* and *C. neoformans*) and bacteria (*E. coli* and *S. aureus*) were mixed and added to sample S11 at three different concentrations (Spike1, Spike2, and Spike3), followed by MinION sequencing. A total of 25,268, 41,049, and 25,609 reads were obtained for Spike1, Spike2, and Spike3, respectively. *N. glabratus* was successfully detected in all tested samples. Specifically, 255 (1.0%), 3,886 (9.47%), and 902 (3.52%) reads were assigned to *N. glabratus* in Spike1, Spike2, and Spike3, respectively (Fig. S1; Table S3). In addition, all mock microbial strains were successfully detected. Reads assigned to *C. albicans* were 1,186 (4.63%), 4,904 (11.95%), and 9,942 (38.82%); those assigned to *C. neoformans* were 39 (0.15%), 145 (0.35%), and 184 (0.72%); those assigned to *E. coli* were 18,343 (71.57%), 17,462 (42.54%), and 8,540 (33.35%); and those assigned to *S. aureus* were 3,018 (11.78%), 11,383 (27.73%), and 4,564 (17.82%) in Spike1, Spike2, and Spike3, respectively. These results further support the efficiency of microbial DNA enrichment and detection from blood culture samples, even in the presence of mixed microbial populations.

### Testing the method

Testing sample S11, MinION sequencing generated 439,989 reads within 3 h, with a mean read length of 3,042 bp and an average read quality score of 14.1 (Fig. S2). Real-time species identification of the first 4,000 reads proposed five species from six genera (Fig. S3). *N. glabratus* was the dominant species represented by 3,449 reads (86.2%), followed by *Sphingobium yanoikuyae* (164 reads; 4.1%), *Niallia taxi* (87 reads; 2.1%), *Ralstonia pickettii* (81 reads; 2.0%), unknown (58 reads; 1.4%), and *Novosphingobium* sp. EMRT-2 (54 reads; 1.3%) (Table S4). No reads were assigned to *Homo sapiens*. Across the complete data set of 439,989 reads, 132 species from 136 genera were identified; only four species had a relative abundance greater than 1%: *N. glabratus*, *S. yanoikuyae*, *N. taxi*, and *R. pickettii*—consistent with results obtained from the first 4,000 reads (Fig. S4; Table S4). Notably, the initial 4,000 reads covered 55.1% of the *N. glabratus* genome with a mean coverage depth of 0.8× (Table S5). These results demonstrate that our protocol enables rapid and accurate species identification within the early stages of sequencing, supporting its utility for real-time diagnosis of fungal BSIs using nanopore technology.

### Real-time species identification by MinION

Next, we performed MinION sequencing on the remaining 47 blood culture samples. The yields of extracted DNA ranged from 0.7  to 36 ng/μL (Table S6). Although the DNA concentrations of samples S2 and S4 were very low before PCR, successful amplification was achieved after PCR. Three independent sequencing runs were conducted using either R9.4 or R10.4 flow cells with multiplexed libraries comprising 11, 24, and 12 samples, respectively. These runs generated 2,380,469, 2,379,064, and 3,027,579 reads, respectively (Table S7).

**Fig 2:**
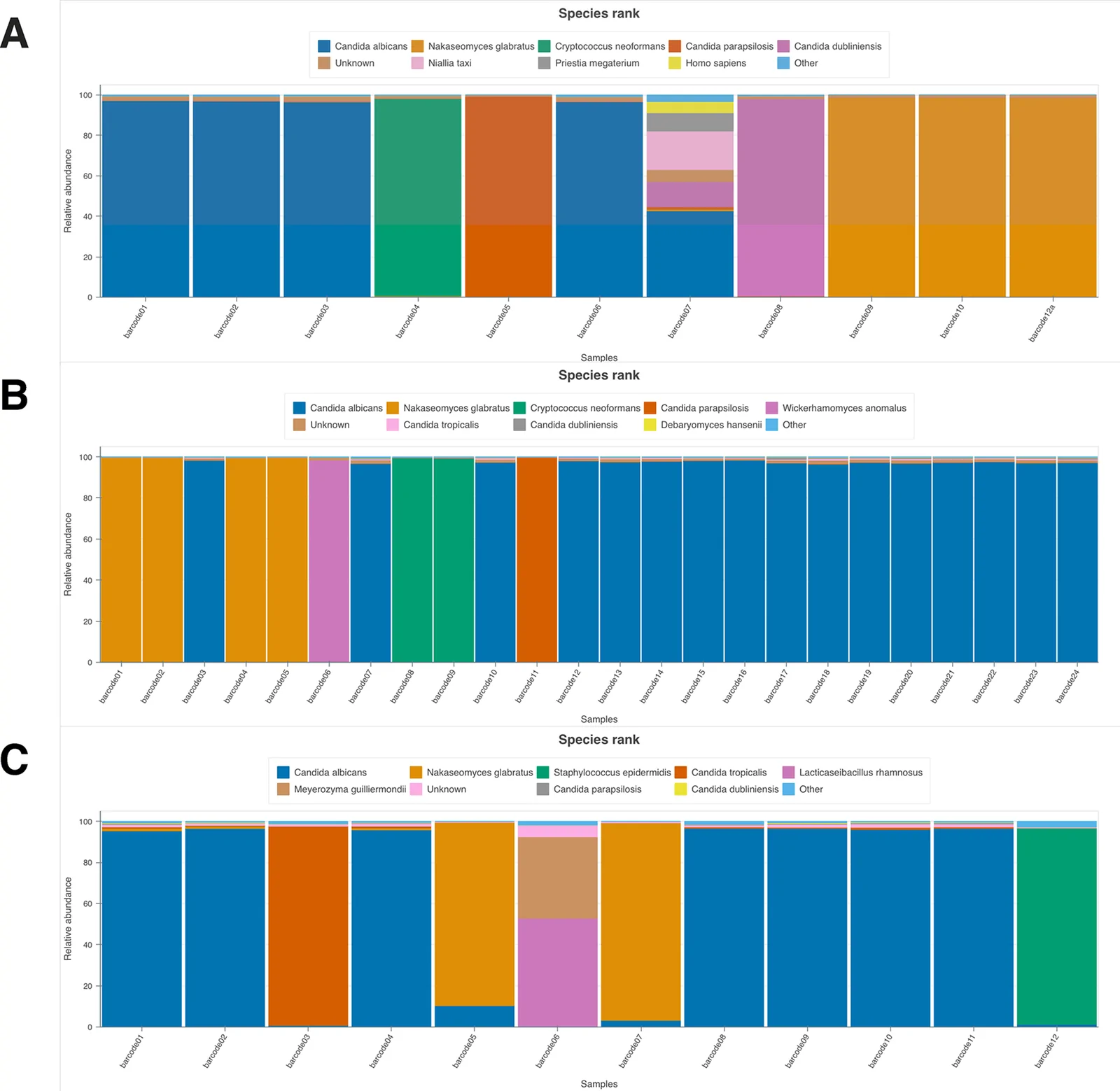
Snapshot of the EPI2ME wf-metagenomics pipeline workflow. (**A**) Relative abundances for MinION sequencing of 11 samples using the R9.4 flow cell. *Candida albicans* and *Candida dubliniensis* were detected in sample S7 labeled with barcode07. This visualization illustrates the real-time taxonomic classification performed during sequencing. (**B**) Relative abundances for MinION sequencing of 24 samples using the R10.4 flow cell. (**C**) Relative abundances for MinION sequencing of 12 samples using the R10.4 flow cell. *Meyerozyma guilliermondii* and *Lacticaseibacillus rhamnosus* were detected in sample S42 labeled with barcode06.

Within the first 1.5 h of sequencing (Fig. 2; Table 3), the numbers of classified reads ranged from 6,329 (S21) to 50,816 (S47) (Tables S8 to S10). In 45 out of 47 samples, more than 80% of reads were assigned to a single species. Among 45 high-confidence cases, 43 species identifications were concordant with the clinical diagnoses. Specifically, 27 out of 28 *C. albicans* BSIs were correctly identified, except for sample S7, in which *C. albicans* was the most abundant species but accounted for 42.1% of the reads. *N. glabratus*, *C. neoformans*, *C. tropicalis*, *C. dubliniensis*, and *W. anomalus* BSIs were correctly classified. In addition, in sample S48, the reads were classified as *S. epidermidis*, whereas the clinical diagnosis was *C. parapsilosis* BSI. Notably, *S. epidermidis* was detected approximately 20 h earlier than *C. parapsilosis* in the clinical diagnosis. These findings suggest that bacterial overgrowth may mask fungal detection in mixed infections, highlighting the applicability of our protocol to both fungal and bacterial BSIs. The proportion of reads assigned to *H. sapiens* ranged from 0% to 5.4%, indicating that human DNA was efficiently depleted across all tested samples.

**TABLE 3:** Early species identification of 47 blood culture samples within the first 1.5 h of MinION sequencing

Sample	Clinical diagnosis	# of totalreads	Most abundant kingdom	Most abundant species	# of reads (% of reads)	# of reads assigned to *H. sapiens* (% of reads)	Flowcell
S1	*C. albicans*	16,000	Fungi	*C. albicans*	15,514 (96.9%)	0 (<0.1%)	R9.4
S2	*C. albicans*	16,000	Fungi	*C. albicans*	15,451 (96.5%)	0 (<0.1%)	R9.4
S3	*C. albicans*	16,000	Fungi	*C. albicans*	15,400 (96.1%)	0 (<0.1%)	R9.4
S4	*C. neoformans*	24,000	Fungi	*C. neoformans*	23,368 (97.3%)	16 (<0.1%)	R9.4
S5	*C. parapsilosis*	16,000	Fungi	*C. parapsilosis*	15,861 (99.1%)	0 (<0.1%)	R9.4
S6	*C. albicans*	16,000	Fungi	*C. albicans*	15,400 (96.2%)	0 (<0.1%)	R9.4
S7	*C. albicans*	16,000	Fungi	*C. albicans*	6,746 (42.1%)	872 (5.4%)	R9.4
S8	*C. dubliniensis*	8,000	Fungi	*C. dubliniensis*	7,751 (96.8%)	0 (<0.1%)	R9.4
S9	*N. glabratus*	8,000	Fungi	*N. glabratus*	7,885 (98.5%)	0 (<0.1%)	R9.4
S10	*N. glabratus*	16,000	Fungi	*N. glabratus*	15,771 (98.5%)	0 (<0.1%)	R9.4
S12	*N. glabratus*	16,000	Fungi	*N. glabratus*	15,778 (98.6%)	0 (<0.1%)	R9.4
S13	*N. glabratus*	7,726	Fungi	*N. glabratus*	7,679 (99.3%)	0 (<0.1%)	R10.4
S14	*N. glabratus*	7,374	Fungi	*N. glabratus*	7,332 (99.4%)	0 (<0.1%)	R10.4
S15	*C. albicans*	10,495	Fungi	*C. albicans*	10,292 (98.0%)	0 (<0.1%)	R10.4
S16	*N. glabratus*	8,692	Fungi	*N. glabratus*	8,622 (99.1%)	0 (<0.1%)	R10.4
S17	*N. glabratus*	9,504	Fungi	*N. glabratus*	9,444 (99.3%)	0 (<0.1%)	R10.4
S18	*W. anomalus*	7,908	Fungi	*W. anomalus*	7,762 (98.1%)	0 (<0.1%)	R10.4
S19	*C. albicans*	23,546	Fungi	*C. albicans*	22,738 (96.5%)	61 (0.2%)	R10.4
S20	*C. neoformans*	8,615	Fungi	*C. neoformans*	8,554 (99.2%)	0 (<0.1%)	R10.4
S21	*C. neoformans*	6,329	Fungi	*C. neoformans*	6,274 (99.1%)	0 (<0.1%)	R10.4
S22	*C. albicans*	9,024	Fungi	*C. albicans*	8,761 (97.0%)	0 (<0.1%)	R10.4
S23	*C. parapsilosis*	8,186	Fungi	*C. parapsilosis*	8,162 (99.7%)	0 (<0.1%)	R10.4
S24	*C. albicans*	10,047	Fungi	*C. albicans*	9,817 (97.7%)	0 (<0.1%)	R10.4
S25	*C. albicans*	10,544	Fungi	*C. albicans*	10,264 (97.3%)	0 (<0.1%)	R10.4
S26	*C. albicans*	8,202	Fungi	*C. albicans*	8,012 (97.6%)	0 (<0.1%)	R10.4
S27	*C. albicans*	38,448	Fungi	*C. albicans*	37,691 (98.0%)	34 (0.1%)	R10.4
S28	*C. albicans*	10,728	Fungi	*C. albicans*	10,524 (98.0%)	0 (<0.1%)	R10.4
S29	*C. albicans*	9,072	Fungi	*C. albicans*	8,770 (96.6%)	0 (<0.1%)	R10.4
S30	*C. albicans*	10,116	Fungi	*C. albicans*	9,731 (96.1%)	0 (<0.1%)	R10.4
S31	*C. albicans*	10,429	Fungi	*C. albicans*	10,126 (97.0%)	0 (<0.1%)	R10.4
S32	*C. albicans*	10,750	Fungi	*C. albicans*	10,391 (96.6%)	0 (<0.1%)	R10.4
S33	*C. albicans*	12,350	Fungi	*C. albicans*	11,987 (97.0%)	0 (<0.1%)	R10.4
S34	*C. albicans*	10,810	Fungi	*C. albicans*	10,534 (97.4%)	0 (<0.1%)	R10.4
S35	*C. albicans*	11,913	Fungi	*C. albicans*	11,525 (96.7%)	0 (<0.1%)	R10.4
S36	*C. albicans*	10,852	Fungi	*C. albicans*	10,526 (96.9%)	0 (<0.1%)	R10.4
S37	*C. albicans*	6,751	Fungi	*C. albicans*	6,422 (95.1%)	23 (0.4%)	R10.4
S38	*C. albicans*	6,763	Fungi	*C. albicans*	6,512 (96.2%)	0 (<0.1%)	R10.4
S39	*C. tropicalis*	9,658	Fungi	*C. tropicalis*	9,303 (96.3%)	14 (0.1%)	R10.4
S40	*C. albicans*	10,653	Fungi	*C. albicans*	10,182 (95.5%)	0 (<0.1%)	R10.4
S41	*N. glabratus*	9,330	Fungi	*N. glabratus*	8,308 (89.0%)	0 (<0.1%)	R10.4
S42	*M. guilliermondii*	7,078	Bacteria	*L. rhamnosus*	3,704 (52.3%)	30 (0.7%)	R10.4
S43	*N. glabratus*	9,082	Fungi	*N. glabratus*	8,715 (95.9%)	0 (<0.1%)	R10.4
S44	*C. albicans*	8,476	Fungi	*C. albicans*	8,164 (96.3%)	0 (<0.1%)	R10.4
S45	*C. albicans*	9,126	Fungi	*C. albicans*	8,785 (96.2%)	0 (<0.1%)	R10.4
S46	*C. albicans*	10,819	Fungi	*C. albicans*	10,362 (95.7%)	0 (<0.1%)	R10.4
S47	*C. albicans*	50,816	Fungi	*C. albicans*	48,981 (96.3%)	0 (<0.1%)	R10.4
S48	*C. parapsilosis*	13,589	Bacteria	*S. epidermidis*	12,903 (94.9%)	12 (<0.1%)	R10.4

### Simultaneous detection of multiple *Candida* species in blood culture

The clinical diagnosis for sample S7 was *C. albicans* BSI. Consistent with the clinical diagnosis, *C. albicans* was the most abundant species, accounting for 6,746 reads (42.1%), followed by *N. taxi* (3,022 reads; 18.8%), *C. dubliniensis* (1,977 reads; 12.3%), *Priestia megaterium* (1,446 reads; 9.0%), unknown (942 reads; 5.8%), and *H. sapiens* (872 reads; 5.4%) (Table S8). Compared with the other BSI samples, the microbial composition of sample S7 suggested a mixed infection pattern (Fig. 2A). The substantial number of reads assigned to *C. dubliniensis* suggests the possibility of co-infection. Notably, BLASTN analysis produced comparable results; *C. albicans* was the dominant species with 6,506 reads (40.7%), followed by *C. dubliniensis* (1,976 reads; 12.4%), *P. megaterium* (1,509 reads; 9.4%), and *H. sapiens* (730 reads; 4.6%) (Table S11).

**Fig 3:**
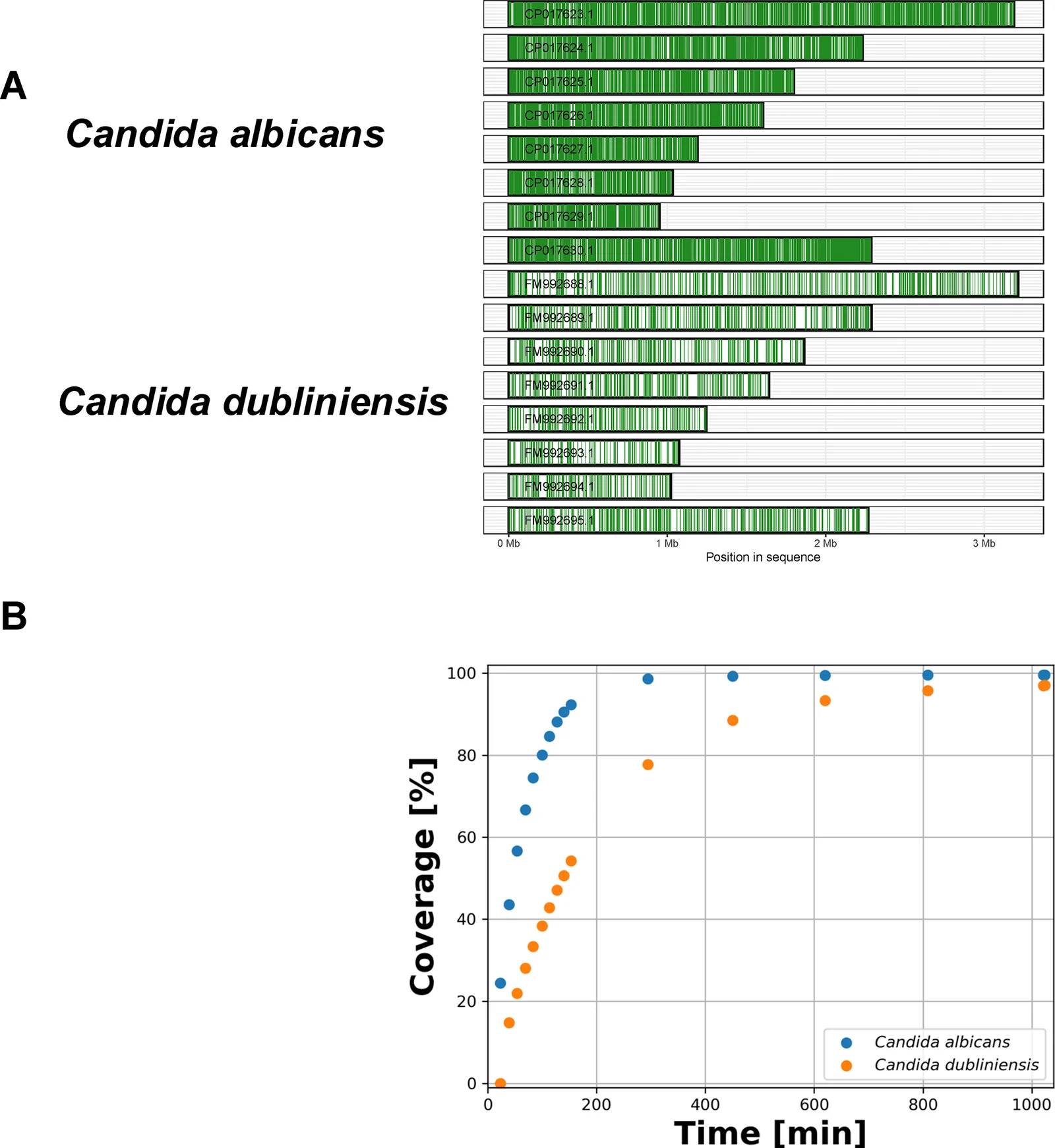
Coverage analysis for *Candida albicans* and *Candida dubliniensis* in sample S7. (**A**) Coverage by the ONT reads. A total of 16,000 reads after the first 1.5 h were mapped to the concatenated genome of *C. albicans* and *C. dubliniensis*. (**B**) Coverage with the sequencing time. The *x* and *y* axes correspond to the sequencing time and the coverage of *C. albicans* (blue) and *C. dubliniensis* (orange).

To further investigate potential co-infection by multiple *Candida* species, the reads from S7 were mapped to a concatenated reference genome of *C. albicans* and *C. dubliniensis*. The reads covered 66.7% of the *C. albicans* genome and 28.0% of the *C. dubliniensis* genome (Fig. 3A). Coverage for both species increased with sequencing time (Fig. 3B; Table S12). Within the first 2.5 h (36,000 reads), 91.7% of the *C. albicans* and 52.2% of the *C. dubliniensis* genomes were covered. After 17 h of sequencing, 243,846 total reads were generated, of which 132,752 reads (54.4%) provided 99.6% coverage of the *C. albicans* genome and 97.1% coverage of the *C. dubliniensis* genome (Fig. S5).

### Simultaneous detection of *Candida* and bacterial species in blood culture

The clinical diagnosis for sample S42 was *M. guilliermondii* BSI. Notably, *Lacticaseibacillus rhamnosus* was also detected during clinical evaluation, suggesting a possible co-infection. Sequencing data supported this finding, with 3,704 reads (52.3%) assigned to *L. rhamnosus* and 2,783 reads (39.3%) to *M. guilliermondii* (Table S8). This taxonomic assignment was validated by BLASTN analysis (Table S13). These results clearly indicate a co-infection and highlight the robustness of our sequencing-based workflow in detecting clinically relevant co-infections directly from blood culture samples.

### Genome-wide mutation profiling of *C. albicans* and *N. glabratus* BSIs

**Fig 4:**
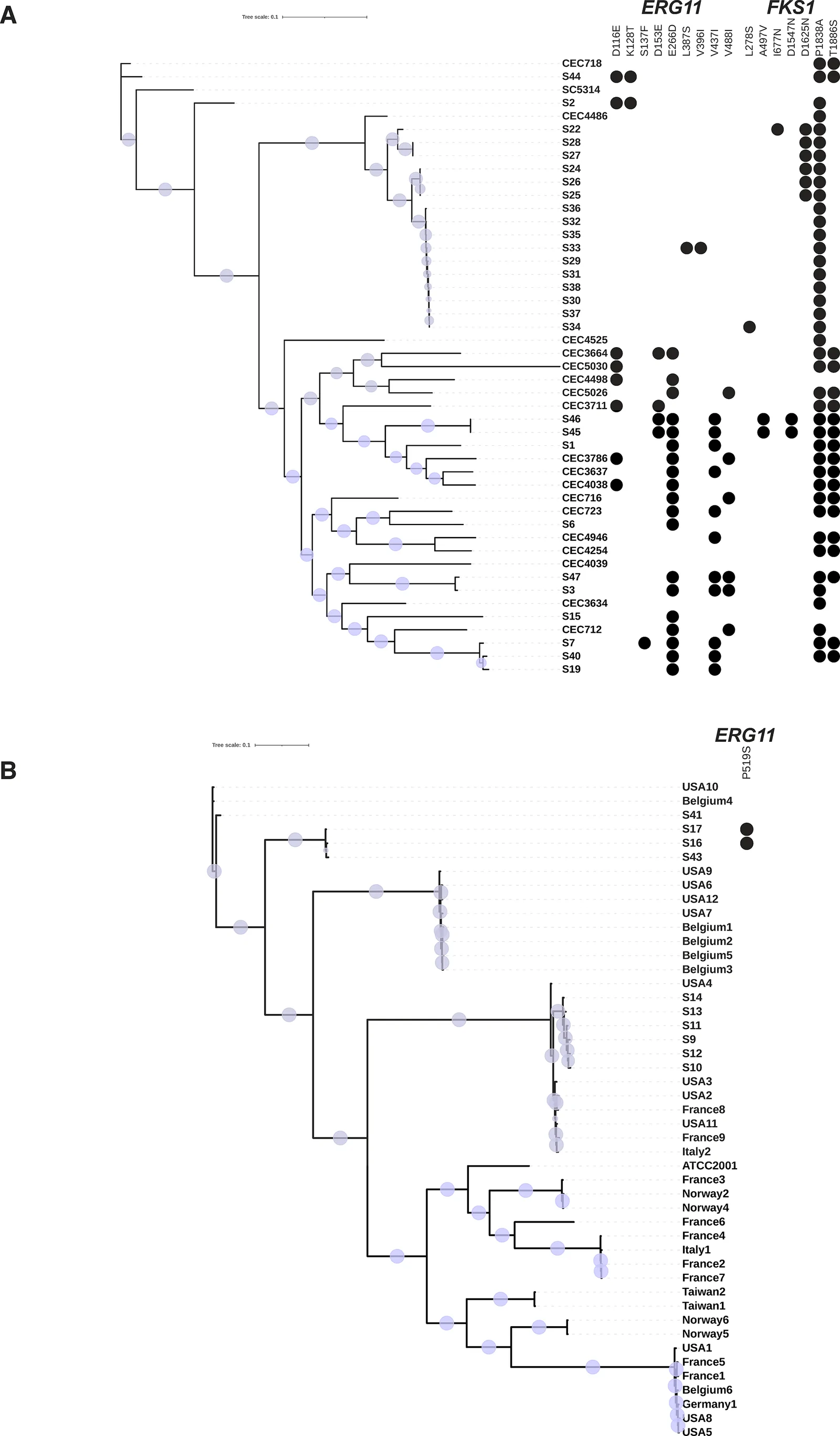
Phylogenetic analyses for *Candida albicans* and *Nakaseomyces glabratus*. (**A**) Phylogenetic tree of 28 *C. albicans* BSI and 18 *C. albicans* isolates. The presence or absence of missense mutations in *ERG11* (D116E, K128T, S137F, D153E, E266D, L387S, V396I, V437I, and V488I) and *FKS1* (L278S, A497V, I677N, D1547N, D1625N, P1838A, and T1886S) detected in the 28 *C. albicans* BSI samples is indicated by black circles. Circle sizes in purple indicate bootstrap support values. (**B**) Phylogenetic tree of 10 *N. glabratus* BSI samples and 38 *N. glabratus* isolates. The presence or absence of missense mutation in *ERG11* (P519S) detected in the 10 *N. glabratus* BSI samples is indicated by black circles.

Our method enables the identification of mutations across the entire genome. To evaluate whether the causative *C. albicans* strains were likely susceptible or resistant to antifungal agents, we analyzed the mutation profiles of *C. albicans* BSIs (*n* = 28). The average number of detected mutations was 136,636 ± 13,701 (Table S14), and the number of SNP differences ranged from 76 between S30 and S38 and 51,390 between S15 and S45 (Table S15). We examined the relationship between sequencing depth and the total number of mutations; however, no clear association was observed, that is, mutation profiles were consistently detected, regardless of sequencing depth (Fig. S6). Missense mutations identified in *ERG11* and *FKS1* (Table S16). Similarly, the mutation profiles of *N. glabratus* BSIs (*n* = 10) were analyzed; and 92,822 ± 2,039 SNPs were detected (Table S17); and the number of SNP differences ranged from 2,750 between S16 and S17 and 88,493 between S11 and S43 (Table S18). Missense mutations identified in *ERG11* but not *FKS1* (Table S19). Finally, phylogenetic analysis based on whole-genome mutations was performed using publicly available genomic data. The 28 *C. albicans* and 10 *N. glabratus* BSI samples were genetically diverse. Among the *C. albicans* isolates, 16 samples clustered with CEC4486, whereas the remaining isolates were widely distributed across the phylogenetic tree (Fig. 4A). Similarly, six *N. glabratus* isolates formed a distinct cluster, while the remaining isolates showed diverse phylogenetic relationships (Fig. 4B). These analyses demonstrated that real-time genomic data can effectively resolve the phylogenetic relationships among individual strains.

## DISCUSSION

In this study, we developed a novel and rapid diagnostic method for fungal BSIs using blood culture samples collected before the culture bottles exhibited a positive signal. The protocol consists of three steps: depletion of human DNA, extraction of fungal DNA, and species identification by PCR and nanopore sequencing using the custom database (Fig. 1). We validated the protocol using a total of 48 clinical blood culture samples and successfully identified *Candida* spp., *Cryptococcus* sp., *Meyerozyma* sp., *Nakaseomyces* sp., and *Wickerhamomyces* sp. We achieved accurate species identification in approximately 7 h starting from DNA extraction. It has been reported that delayed initiation of antifungal therapy, particularly beyond 72 h after the initial positive blood culture is drawn, is associated with a trend toward lower patient survival (50). By applying our protocol at 24 h after incubation, fungal pathogens could potentially be identified within approximately 30 h in total, including sequencing and analysis time. This reduction in diagnostic turnaround time may contribute to improved clinical outcomes by enabling earlier initiation of appropriate antifungal therapy, highlighting the potential of our approach to support earlier and more effective antifungal treatment in clinical settings. Benzonase treatment was effective in reducing human DNA in contrast to the study in which only saponin treatment was applied to blood culture samples for PCR-based bacterial detection (51). Importantly, our protocol requires only 1 mL of blood culture broth, allowing for integration into standard clinical workflows without the need for additional blood sampling. Therefore, periodic sampling during incubation could enable sequential monitoring and earlier pathogen detection. This makes the method clinically feasible and compatible with existing workflows. Furthermore, it can be conducted in parallel with the current gold standard methods, offering a complementary and rapid diagnostic tool for early pathogen detection and intervention.

Spike-in experiments validated that our protocol is suitable for blood culture samples containing mixed infections (Fig. S1; Table S3). However, only a small number of reads were assigned to *C. neoformans*. This may be due to a lower number of fungal cells in the sample or reduced DNA extraction efficiency compared with the other microorganisms tested. Nevertheless, all *C. neoformans* BSI samples were successfully detected, indicating that the workflow is capable of identifying this pathogen in clinical specimens.

In the case of sample S7, we detected a potential fungal co-infection involving *C. albicans* and *C. dubliniensis. C. dubliniensis* may have co-infected the patient alongside *C. albicans*. The ACT1 open reading frame shares 98% nucleotide sequence identity between *C. albicans* and *C. dubliniensis* (52), which may lead to misidentification in clinical diagnostics. This potential co-infection highlights the utility of comprehensive metagenomic sequencing in uncovering fungal co-infections that might otherwise go undetected. Recognizing the co-infections could be valuable for clinical decision-making, particularly in selecting appropriate antifungal therapy.

In the case of sample S42, we identified a fungal and bacterial co-infection caused by *M. guilliermondii* and *L. rhamnosus*. This finding supports the effectiveness of our DNA extraction protocol, which successfully enriched both fungal and bacterial DNA. In contrast, only *S. epidermidis* was detected in sample S48 (Table S10), with no *C. parapsilosis* reads identified, suggesting that the timing of blood culture sampling may influence the detectability of fungal pathogens. Moreover, a high abundance of bacterial DNA may mask the presence of fungal species during sequencing analysis. Collectively, these observations highlight the need for careful optimization of both blood collection timing and sequencing workflows to ensure high diagnostic sensitivity.

Furthermore, the sequencing data can be utilized to detect missense mutations in specific genes, thereby enabling the prediction of whether the causative strains are resistant or susceptible to antifungal agents (Fig. 4; Tables S16 and S19). No mutations associated with azole resistance, including *ERG11* A114S, Y132H, Y132F, K143R, Y257H, and K143Q in *C. albicans*, were identified (53, 54). In addition, among the tested samples, no mutations associated with elevated micafungin and caspofungin minimum inhibitory concentrations (MICs), such as *FKS1* S645P in *C. albicans* and *FKS1* S629P in *N. glabratus*, were found (55, 56). The prior estimation of antifungal drug susceptibility in causative strains could assist in guiding subsequent therapy and improving clinical outcomes.

Our method offers several key advantages: high accuracy in species-level identification, broad-spectrum detection across diverse fungal and bacterial taxa, and effective depletion of human DNA, thereby improving diagnostic resolution. To our knowledge, this represents the first successful diagnostic protocol enabling species-level identification directly from blood culture before positivity is flagged in automated blood culture systems. Notably, our method accurately identified a wide array of pathogens encompassing both fungal and polymicrobial BSIs. The detection of *S. epidermidis* and *L. rhamnosus* suggests potential applicability for bacterial BSI diagnostics as well. Importantly, this rapid and comprehensive diagnostic platform can enable early and targeted administration of antifungal therapy, which is critical for reducing mortality in patients with fungal BSIs. Our findings highlight the potential of this approach to uncover previously undiagnosed co-infections, providing a more complete picture of the infectious landscape in critically ill patients.

Nevertheless, several limitations of this study should be acknowledged. First, the optimal incubation time required for reliable fungal detection has not yet been fully established. Additional studies will be required to determine the most effective sampling time points during blood culture incubation. Second, in cases of bacterial co-infection, rapid bacterial proliferation may mask fungal growth and reduce the sensitivity of fungal pathogen detection. This phenomenon was also observed in some clinical samples analyzed in the present study. Therefore, further optimization of the workflow and validation using larger cohorts of mixed-infection samples will be necessary to improve robustness and clinical applicability.

## Data Availability

Raw reads have been deposited in the DDBJ BioProject database (BioProject accession number PRJDB42230).

## SUPPLEMENTAL MATERIAL

Supplemental figures spectrum.00478-26-s0001.pdf Fig. S1 to S6.

Supplemental tables spectrum.00478-26-s0002.xlsx Tables S1 to S19.

ASM does not own the copyrights to Supplemental Material that may be linked to, or accessed through, an article. The authors have granted ASM a non-exclusive, world-wide license to publish the Supplemental Material files. Please contact the corresponding author directly for reuse.
