# Supplementary material for: Random PCR-based nanopore whole-genome sequencing enables pre-positivity detection of fungal bloodstream infections
Source: Microbiol Spectr. 2026 Aug 21;14(10):e00478-26. doi: 10.1128/spectrum.00478-26 (PMC13688911; doi:10.1128/spectrum.00478-26)
Supplement: Supplementary file 1 [file spectrum.00478-26-s0001.pdf]

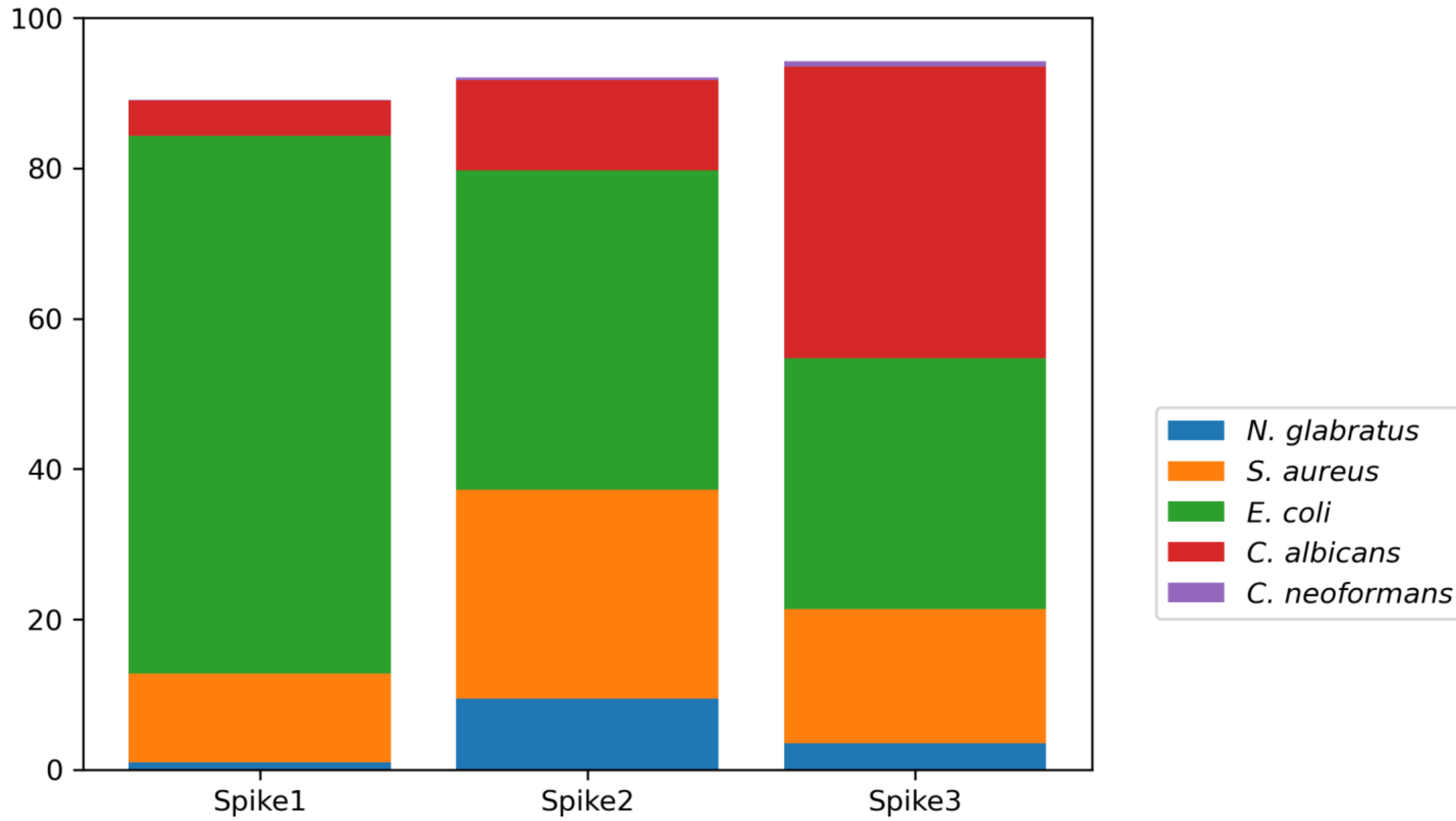

Figure S1. Results of spike-in validation experiments.
Relative abundances of microbial species detected by MinION sequencing in Spike1, Spike2, and Spike3. *N. glabratus* and all mock microbial strains (*C. albicans*, *C. neoformans*, *E. coli*, and *S. aureus*) were successfully detected in all spike-in conditions. Spike1: OD_{600} = 5 for both fungi and bacteria; Spike2: OD_{600} = 1 for both fungi and bacteria; Spike3: fungi OD_{600} = 5 and bacteria OD_{600} = 1.

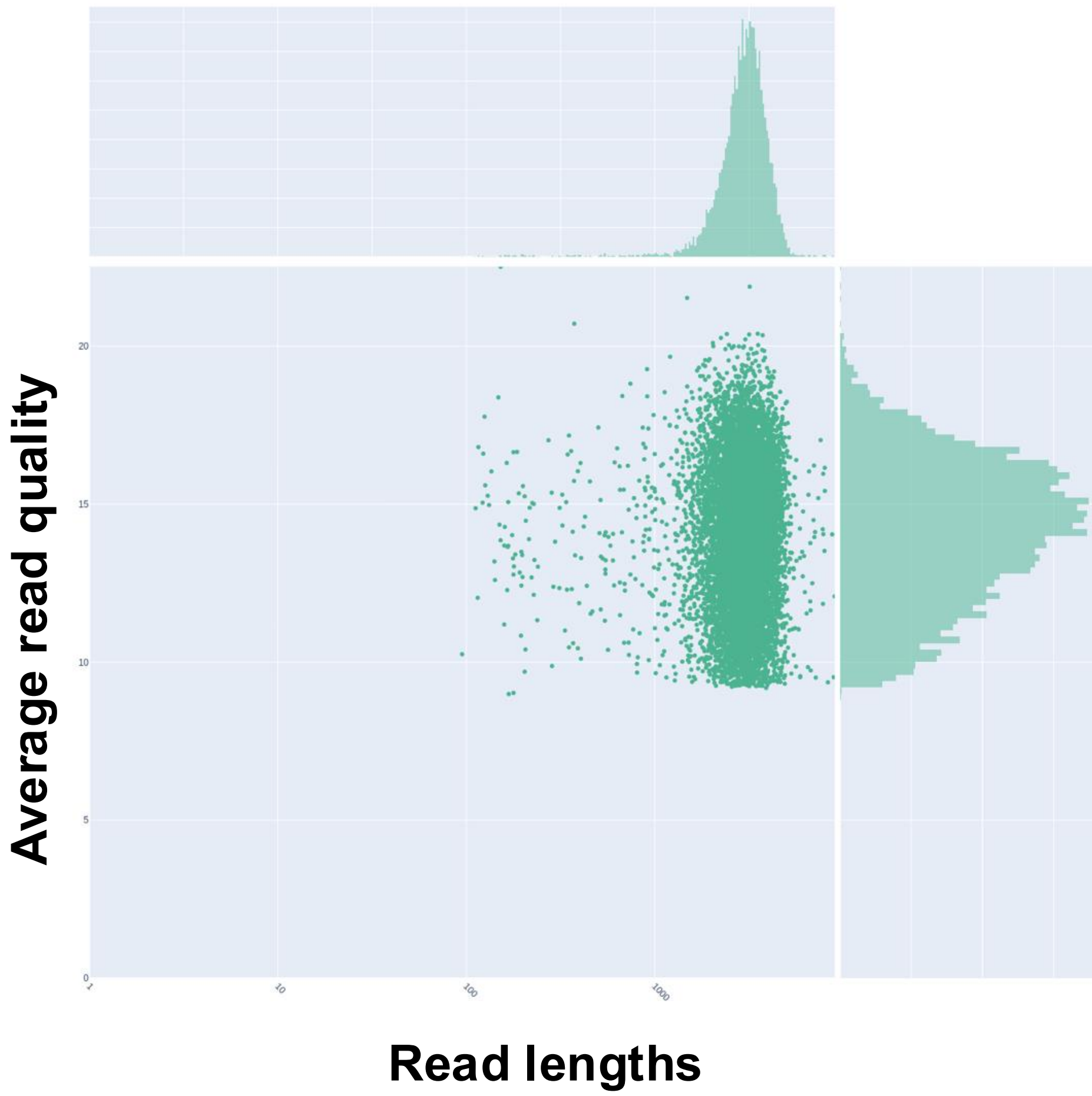

Figure S2. MinION sequencing quality of sample S11.
The *x* and *y* axes represent read length (bp) and average read quality, respectively.

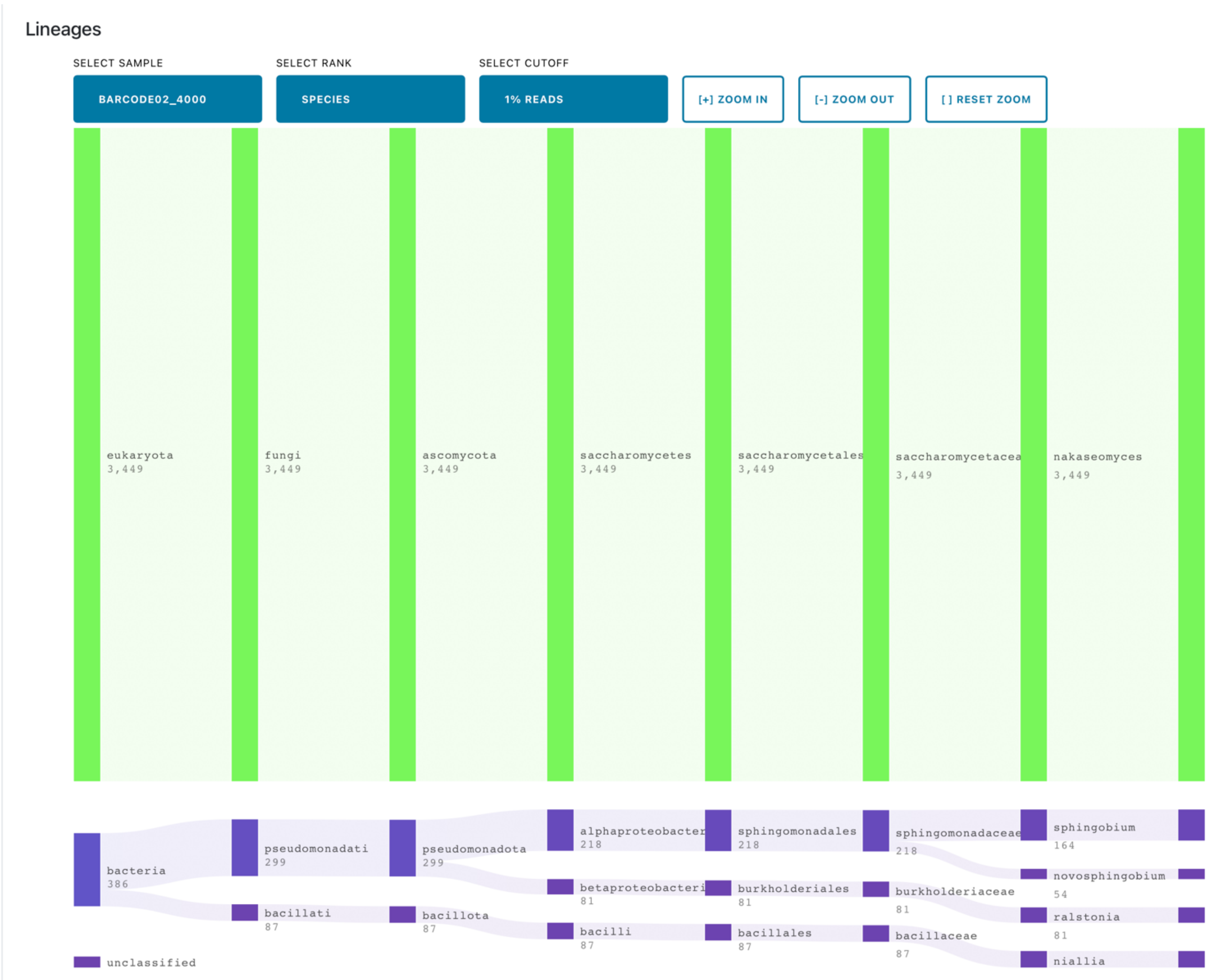

Figure S3. Snapshot of the EPI2ME wf-metagenomics pipeline workflow in sample S11. Among 4,000 reads, 3,449 reads (86.2%) were assigned to *Nakaseomyces glabratus*.

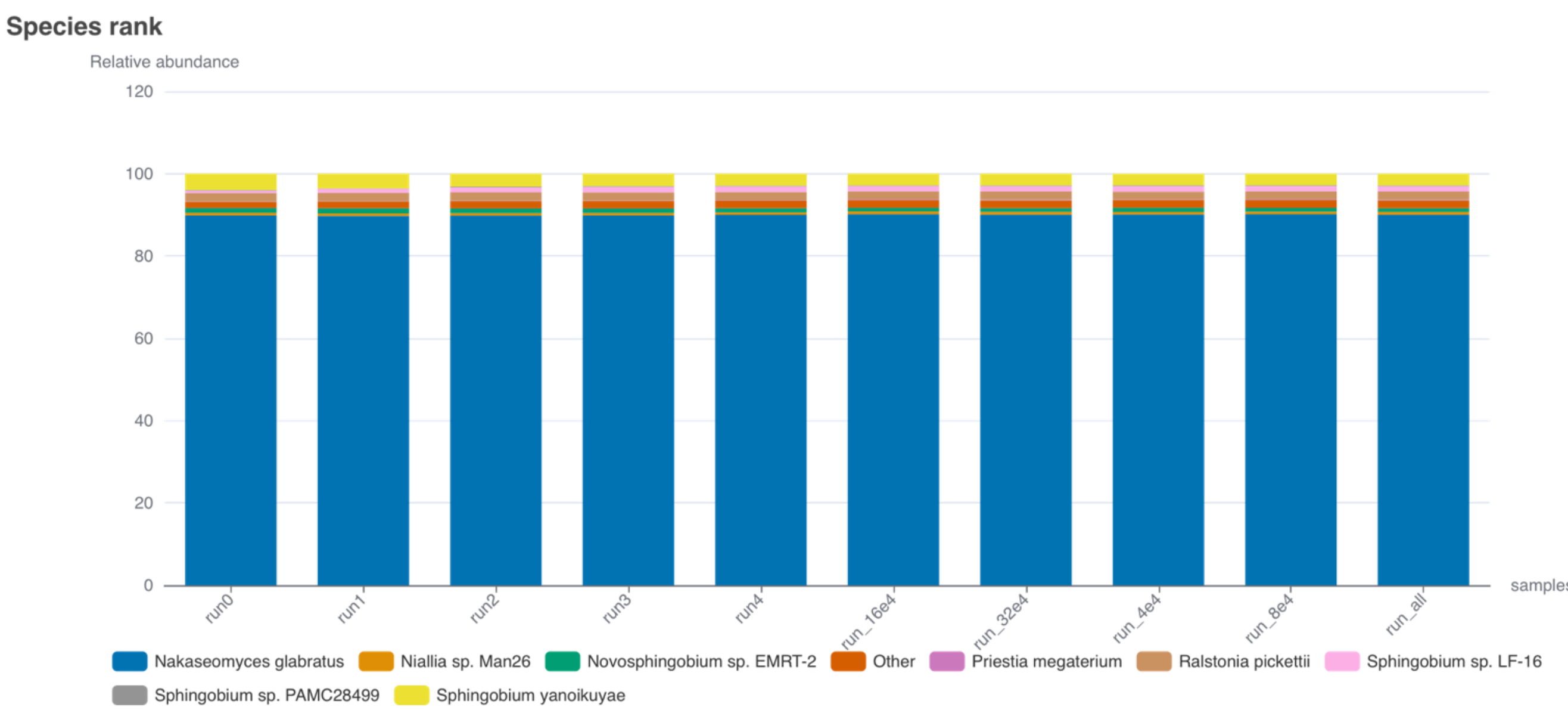

Figure S4. Snapshot of the EPI2ME wf-metagenomics pipeline workflow in sample S11. The bar plots labeled with run0, run1, run2, run3, run4, run_4e4, run_8e4, run_16e4, run_32e4, run_all correspond to analyses using for 4,000, 8,000, 12,000, 16,000, 20,000, 40,000, 80,000, 160,000, 320,000, and 439,989 reads.

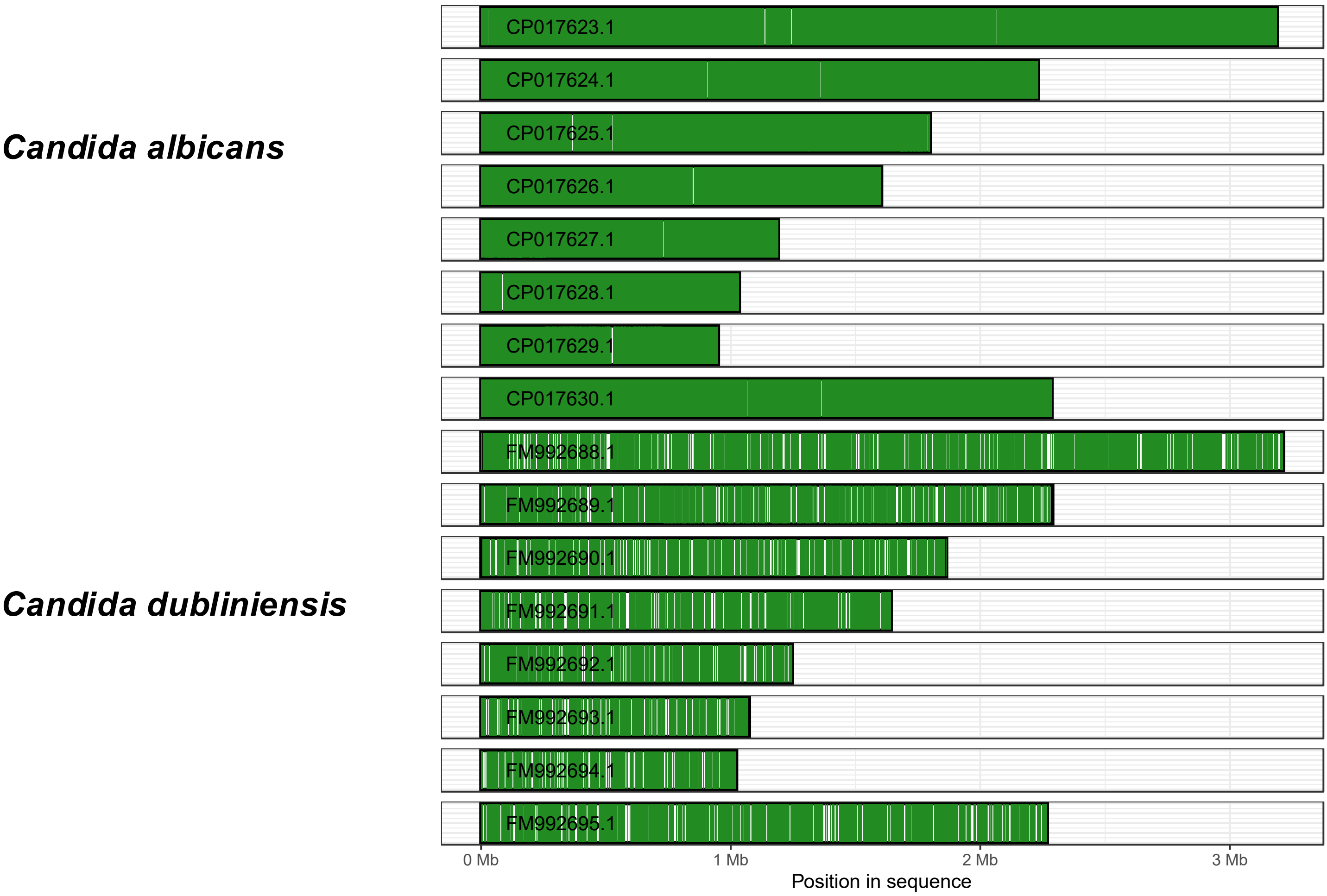

Figure S5. The S7 coverage analysis for *Candida albicans* and *Candida dubliniensis*. A total of 243,846 reads were mapped to the concatenated genome of *C. albicans* and *C. dubliniensis*.

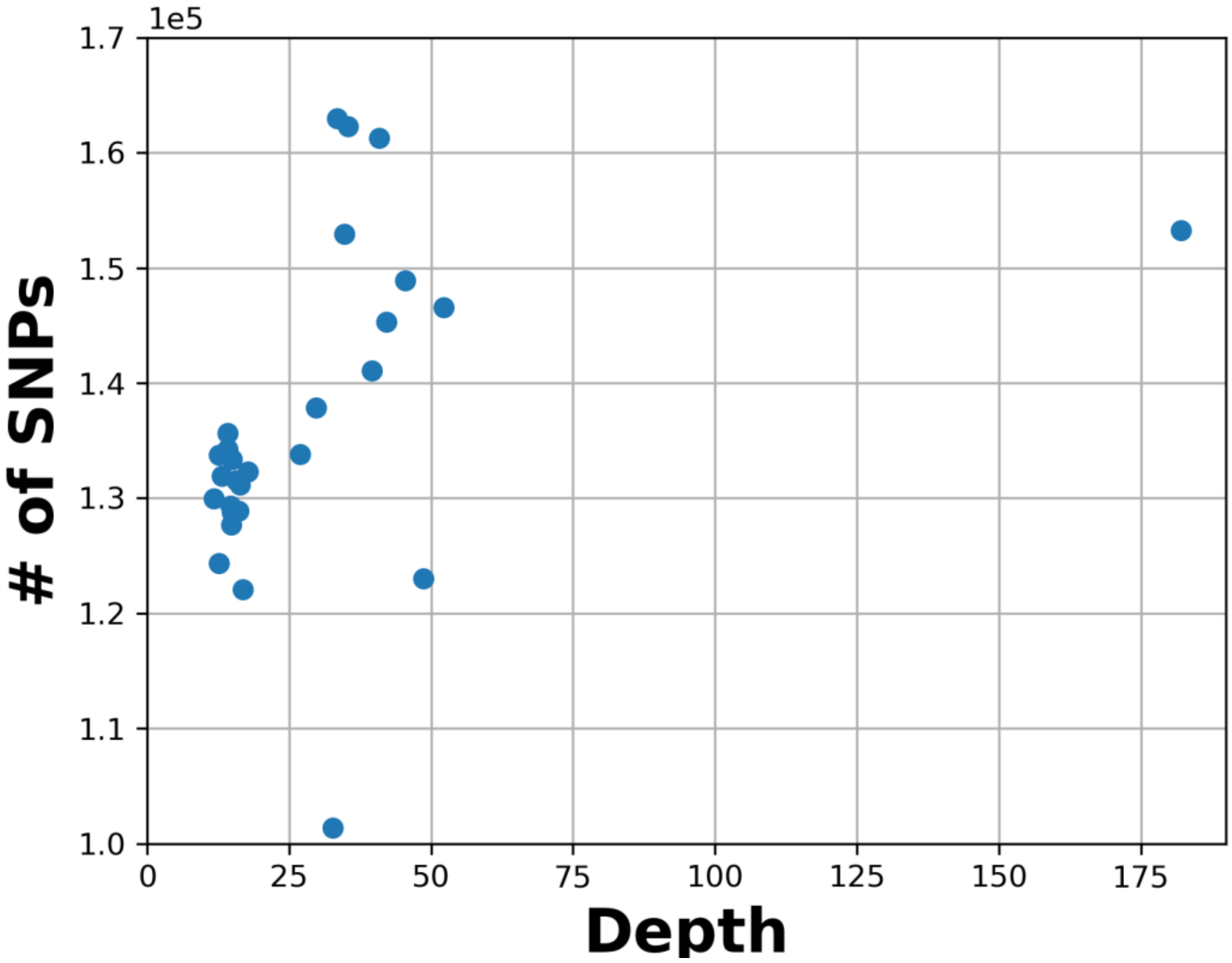

Figure S6. SNP analysis in 28 *Candida albicans* BSI samples.
The *x* and *y* axes represent sequencing depth and the number of SNPs detected, respectively.
